# 6-Aza-2-Thio-Thymine Stabilized Gold Nanoclusters as Photoluminescent Probe for Protein Detection

**DOI:** 10.3390/nano10020281

**Published:** 2020-02-07

**Authors:** Hao-Hua Deng, Xiao-Qiong Shi, Paramasivam Balasubramanian, Kai-Yuan Huang, Ying-Ying Xu, Zhong-Nan Huang, Hua-Ping Peng, Wei Chen

**Affiliations:** Fujian Key Laboratory of Drug Target Discovery and Structural and Functional Research, School of Pharmacy, Fujian Medical University, Fuzhou 350004, China; DHH8908@163.com (H.-H.D.); xiaoqiongshi@163.com (X.-Q.S.); chembala55@gmail.com (P.B.); 15980269577@163.com (K.-Y.H.); yingyingxu2010@hotmail.com (Y.-Y.X.); hzn651806692@163.com (Z.-N.H.); phpfjmu@126.com (H.-P.P.)

**Keywords:** gold nanocluster, 6-aza-2-thio-thymine, protein, fluorescence

## Abstract

This study puts forward an efficient method for protein detection in virtue of the tremendous fluorescence enhancement property of 6-aza-2-thio-thymine protected gold nanoclusters (ATT-AuNCs). In-depth studies of the protein-induced photoluminescence enhancement mechanism illustrate the mechanism of the interaction between ATT-AuNCs and protein. This new-established probe enables feasible and sensitive quantification of the concentrations of total protein in real samples, such as human serum, human plasma, milk, and cell extracts. The results of this proposed method are in good agreement with those determined by the classical bicinchoninic acid method (BCA method).

## 1. Introduction

Proteins are the basic material of living organisms. There have been various methods for the accurate measurement of protein content, which are indispensable tools for biological researches, such as proteomics, molecular biology, cell biology, biochemistry, and neuroscience [1,2,3,4]. However, these methods have their own scope of application and limitations. Therefore, the development of a universal, precise, and rapid quantitative technique will provide a great deal of impetus for the molecular biology revolution and promote the progress of clinical diagnosis.

Owing to their unique physical and chemical properties caused by quantum confinement effect, gold nanoclusters (AuNCs) have emerged as an intensely pursued material for nanoscience research and biological applications in the past decade [5,6,7,8,9]. The molecule-like properties of AuNCs, such as discrete electronic states and size-dependent photoluminescence, makes them a distinguished material from larger nanoparticles and bulk metals [10,11,12,13,14,15,16,17,18]. As a matter of fact, the gradually extensive application of few-atom metal nanomaterials has promoted a depth exploration of nanotoxicology, and, correspondingly, highlighted the significance for the biosafety evaluation to be performed. Investigations of protein–nanoparticle interactions have been reported in several studies [19,20,21,22,23]. However, as far as we know, there has been a smaller number of literatures focusing on protein determination with the help of fluorescent AuNCs [24,25].

In our previous study, we found that the restriction of ligand motion can enhance the fluorescence quantum yield of 6-aza-2-thio-thymine protected gold nanoclusters (ATT-AuNCs) [8,26]. Inspired from this fact, we here test the influence of protein absorption on the fluorescence characteristics of ATT-AuNCs. The interaction between protein and ATT-AuNCs results in a dramatical enhancement on the fluorescence intensity of AuNCs. Based on this, we designed a universal method for detecting total protein (bovine serum albumin) in biological samples.

## 2. Materials and Methods

### 2.1. Chemical and Reagents

6-Aza-2-thiothymine (ATT) was purchased from Alfa Aesar Chemicals Co. Ltd. (Beijing, China). HAuCl_4_·3H_2_O was obtained from Aladdin Reagent Company (Shanghai, China). Bovine serum albumin (BSA) and NaOH was bought from Sinopharm Chemical Reagent Co. Ltd. (Shanghai, China). BCA kit was bought from Solarbio Science and Technology Co. Ltd. (Beijing, China). All chemicals and solvents were of analytical grade and commercially available. All solutions were prepared with deionized water (DIW).

### 2.2. Instruments

UV-2450 UV-Vis spectrophotometer (Shimadzu, Japan) and microplate reader (BioTek Instruments, Winooski, VT, USA) were used for UV-Vis measurements. The fluorescence spectra were recorded on a Cary Eclipse fluorescence spectrophotometer (Agilent, Santa Clara, CA, USA).

### 2.3. Preparation of ATT-AuNCs

Fluorescent ATT-AuNCs were prepared with a facile one-pot strategy as described previously [8]. In short, ATT (15 mL, 80 mM) containing 0.2 M NaOH was added into an aqueous solution of HAuCl_4_ (15 mL, 10 mg/mL), and the mixture was continuously stirred for 1 h at room temperature. The ATT-AuNCs were purified by ultrafiltration (Millipore, Bedford, MA, USA, 50 kDa) and stored at 4 °C in the dark prior to use.

### 2.4. Sample Analysis

In a typical experiment, (a) to 200 μL of ATT-AuNCs stock solution (0.5 mg/mL, pH 5), 20 μL of different concentrations of BSA was added; the mixture was incubated at 33 °C for 25 min; the resulting reaction solution was measured by using fluorescence spectrometer under the excitation wavelength of 472 nm. (b) To 200 μL of BCA stock solution, 20 μL of different concentrations of BSA was added; the mixture was incubated at 37 °C for 25 min; the resulting reaction solution was measured by using a BioTek Microplate reader at 562 nm absorbance wavelength.

Human plasma and serum samples were donated by the Affiliated First Hospital of Fujian Medical University (Fuzhou, China). Our project has been approved by the Medical Ethics Committee of Fujian Medical University. We have obtained informed consent before the experiment, and the information of the volunteers was kept confidential. Milk samples were purchased from local supermarket. All samples were directly used for analysis.

HL60 cells were obtained from Shanghai Institute of Cell Biology and Biochemistry (Shanghai, China) and grown in RPMI 1640 medium (Hyclone) supplemented with 10% fetal calf serum.

For protein determination in plasma and serum samples, the samples were simply diluted by DIW three times, and determined according to the processes mentioned above. Standard addition experiments were further conducted by adding three different concentrations of BSA in the real plasma samples.

For protein determination in milk samples, the samples were simply diluted by DIW about three times. After completion of the reaction, 3 mM of EDTA was added to each sample to eliminate the possible interference of Ca^2+^, and then determined according to the processes mentioned above.

For protein determination in cell extracts, HL60 cells were centrifuged and resuspended in 0.9% NaCl solution. Then, collected HL 60 cells were lysed by lysing buffer (50 mM Gly-NaOH, 0.15 M NaCl, 1% Triton X-100, 1 mM EDTA, 0.1% SDS). Cell extracts were collected by centrifugation, and then simply diluted with DIW three times. Protein content was determined according to the processes mentioned above. Results of the proposed method were also compared with the classical bicinchoninic acid (BCA) method.

## 3. Results and Discussion

### 3.1. Interaction between Protein and ATT-AuNCs

**Figure 1:**
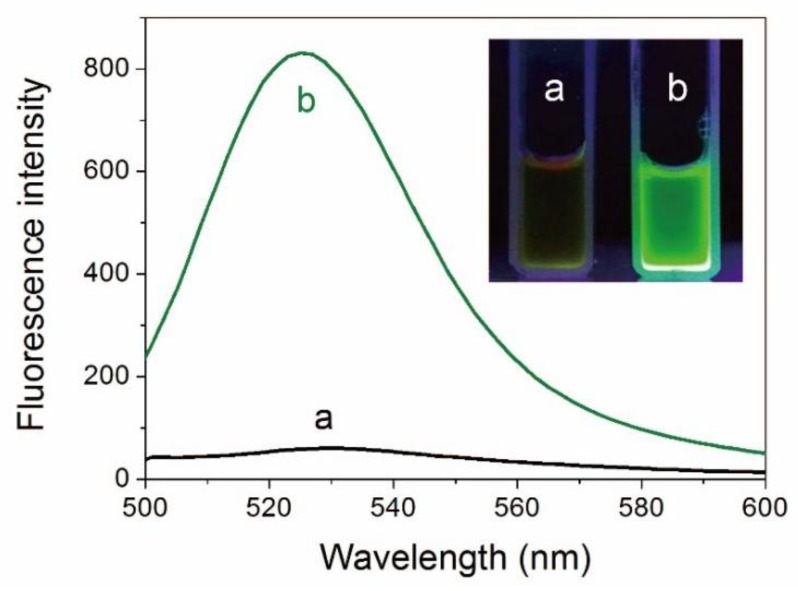
Fluorescence spectra of 6-aza-2-thio-thymine protected gold nanoclusters (ATT-AuNCs) (**a**) before and (**b**) after introducing bovine serum albumin (BSA). The concentrations of ATT-AuNCs and BSA were 0.5 mg/mL and 500 μg/mL, respectively. Inset: Photographs of ATT-AuNC and BSA/ATT-AuNC solutions under UV light.

AuNCs were prepared via a simple one-pot strategy employing ATT as the reducing-cum-stabilizing/capping ligand [8]. The ATT-AuNCs in aqueous solution had a fluorescence emission band centered on 528 nm (photoluminescence quantum yield = 1.8%). As shown in Figure 1, interaction between BSA and ATT-AuNCs provokes a substantial enhancement (13.8-fold) of fluorescence intensity. This remarkable fluorescence change can be obviously observed by the naked eye under the irradiation of UV light (Figure 1 inset). Since BSA has no fluorescence in this spectral region, the enhancement of fluorescence can be reasonably attributed to the adsorption of ATT-AuNCs on the protein, which results in the restriction of rotation and vibration of the ligands.

**Figure 2:**
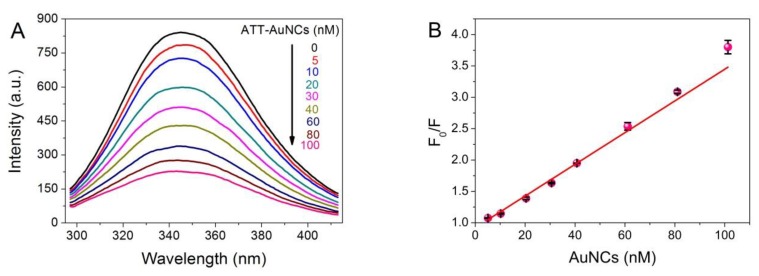
(**A**) Photoemission spectra of BSA (7.5 × 10^−7^ M, 50 μg/mL) with different concentrations of ATT-AuNCs (range from 0 to 100 nM) upon excitation at 280 nm at pH 5.0, 33 °C. (**B**) Stern–Volmer plot for fluorescence quenching of BSA by ATT-AuNCs. Error bars represent the standard deviations across three repetitive experiments.

Fluorescence spectroscopy is a most widely used experimental approach to obtain local conformational or dynamic changes of protein, in which tryptophan (Trp) and tyrosine (Tyr) are the main contributors to the endogenous fluorescence [27]. To investigate the interaction between ATT-AuNCs and BSA, fluorescence quenching of BSA upon the addition of ATT-AuNCs was performed. The photoemission intensity of BSA at 345 nm was suppressed progressively with increasing ATT-AuNCs concentration (Figure 2). Data were then analyzed with the Stern–Volmer equation (Equation (1)).

F_0_/F = 1 + K_q_τ_0_[Q] = 1 + K_sv_[Q] (1)

where F_0_ and F represent the fluorescence intensities of BSA at 345 nm before and after the addition of AuNCs, respectively; [Q] is the concentration of AuNCs; K_sv_ is the Stern–Volmer fluorescence quenching constant; and K_q_ is the bimolecular dynamic fluorescence quenching rate constant. τ_0_ is the lifetime of BSA without the addition of AuNCs, and is known to be approximately 5 × 10^−9^ s. From linear regression of the plot (r = 0.9987), K_q_ = 5.05 × 10^15^ L mol^−1^ s^−1^ was obtained. As the maximum value of K_q_ for a diffusion-controlled quenching process is about 2.0 × 10^10^ L mol^−1^ s^−1^, we concluded a static quenching mechanism was responsible for the quenching process of BSA caused by ATT-AuNCs [28].

The forces of interaction between BSA and ATT-AuNCs were also investigated. Thermodynamic parameters were obtained from the static quenching equation (Equation (2)) and van ’t Hoff equation (Equation (3))

lg[(F_0_ − F)/F] = lgK + nlg[Q] (2)

ΔG = −RTlnK = ΔH − TΔS
(3)

**Figure 3:**
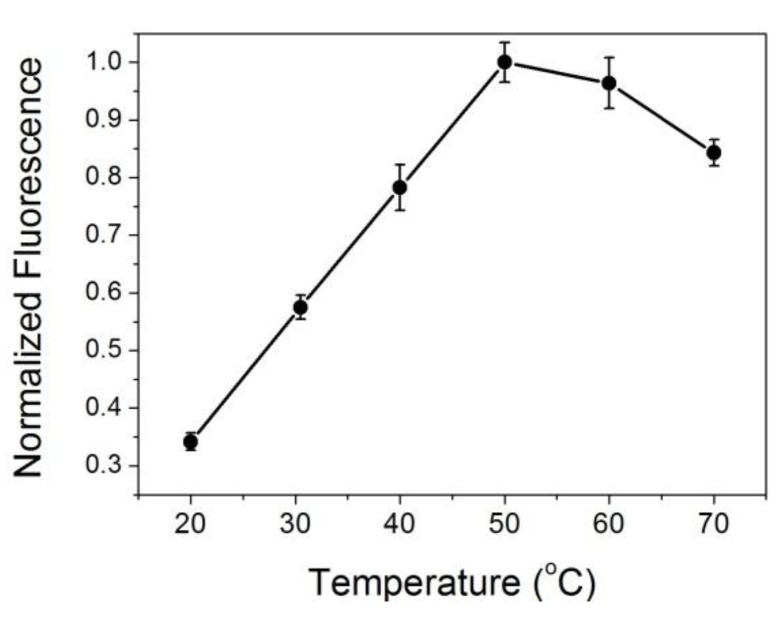
The thermodynamics curve of ATT-AuNCs (8 μM) after introducing BSA (35 μg/mL). Error bars represent the standard deviations across three repetitive experiments.

As can be seen from Table 1, the negative ΔG values suggest that the interaction between ATT-AuNCs and BSA is spontaneous. The positive ΔH and ΔS values indicate that the interaction between ATT-AuNCs and BSA is mainly hydrophobic forces. It might be that when ATT-AuNCs are close to the binding sites of BSA, the hydration layer of BSA around the binding sites are partly destroyed, leading to heat absorption phenomenon [25]. We also found that the photoluminescence intensity of ATT-AuNCs was enhanced as the temperature increased in a certain range, which further validated that ATT-AuNCs approaching the binding sites of target protein is an endothermic reaction (Figure 3).

### 3.2. Analytical Performance

**Figure 4:**
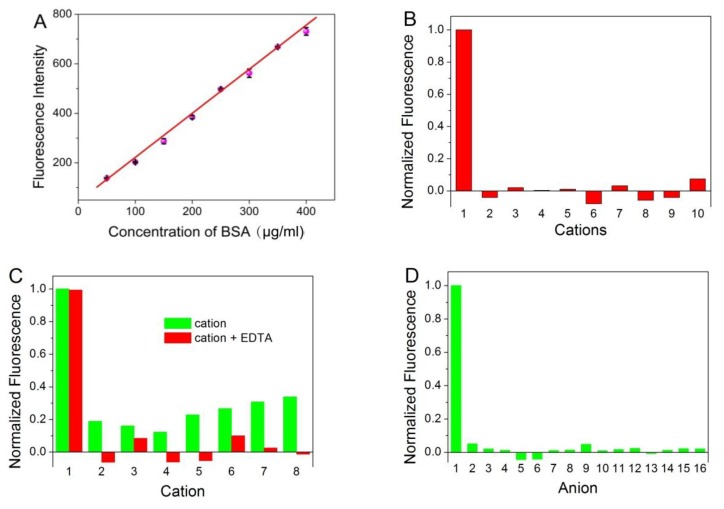

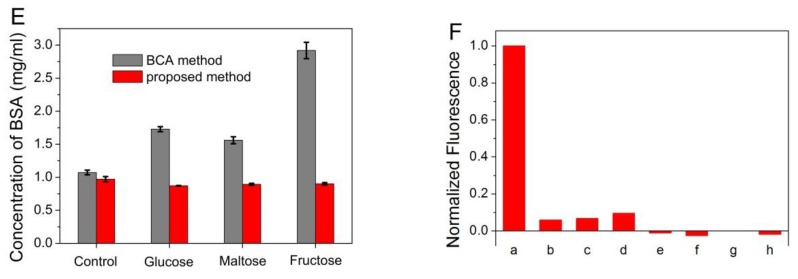
(**A**) The plot of the fluorescence intensity of ATT-AuNCs versus the concentration of BSA. Error bars represent the standard deviations across three repetitive experiments. (**B**) Fluorescence response of ATT-AuNCs to different cations. Samples marked 1 to 10 correspond to BSA (250 μg/mL), Ag^+^, Cr^3+^, K^+^, Na^+^, Hg^2+^, NH_4_^+^, Fe^3+^, Cu^2+^, and Mn^2+^, respectively. The concentration of each cation was 2 mM. (**C**) Fluorescence response of ATT-AuNCs to different cations in the presence of 2.5 mM of EDTA. Samples marked 1 to 8 correspond to BSA (250 μg/mL), Ni^2+^, Ba^2+^, Pb^2+^, Zn^2+^, Mg^2+^, Ca^2+^, Al^3+^, respectively. The concentration of each cation was 2 mM. (**D**) Fluorescence response of ATT-AuNCs to different anions. Samples marked 1 to 16 correspond to BSA (250 μg/mL), HCOO^−^, NO_2_^−^, NO_3_^−^, CN^−^, IO_3_^−^, PO_4_^3−^, SO_4_^2−^, Br^−^, SCN^−^, I^−^, BrO_3_^−^, ClO_4_^−^, F^−^, Cl^−^, and CO_3_^2−^, respectively. The concentration of each anion was 1 mM. (**E**) The detection results of BSA in the presence of different reducing sugar (5 M). Error bars represent the standard deviations across three repetitive experiments. (**F**) Fluorescence response of ATT-AuNCs to (a) 250 μg/mL BSA, (b) 0.5% SDS, (c) 20 mM creatine, (d) 0.5% Tween 20, (e) 150 mM urea, (f) 5% ethanol, (g) 20 mM EDTA, and (h) 0.5% TritonX-100. The normalized fluorescence intensity is calculated by (I − I_0_)/(I_BSA_ − I_0_), where I_0_, I, and I_BSA_ is the fluorescence intensity of ATT-AuNCs, ATT-AuNCs + interferent, ATT-AuNCs + BSA, respectively.

Fluorescence spectrophotometer was used to evaluate the BSA concentration in aqueous solution. In can be seen from Figure 4A, a progressive enhancement of photoluminescence intensity was obtained with the increased amount of BSA. The value of intensity exhibited a good linear correlation to BSA concentration in the range from 50 to 400 μg/mL (r = 0.9955). The detection limit of BSA was 0.88 μg/mL at an S:N ratio of 3. The relative standard deviation is 2.25% for the determination of 200 μg/mL BSA (n = 10).

In order to examine the selectivity of the established protein assay, interferences from common ions were investigated. In addition, certain compounds often used during protein purification or cell lysis were also investigated. Figure 4B,D reveals that ATT-AuNCs were specific toward BSA over the tested cations or anions. Some of the tested cations that interfere with the protein assay can be eliminated to a certain extent by adding the chelator EDTA (Figure 4C). Figure 4E reveals that this gold nanoprobe based on PL enhancement exhibits the remarkable merits of tolerating reducing sugar, compared with the BCA method which gives false positive results. Figure 4F reveals that certain compounds often used during protein purification or cell lysis have little effect on the system.

### 3.3. Determination of Total Protein in Human Plasma and Serum

**Figure 5:**
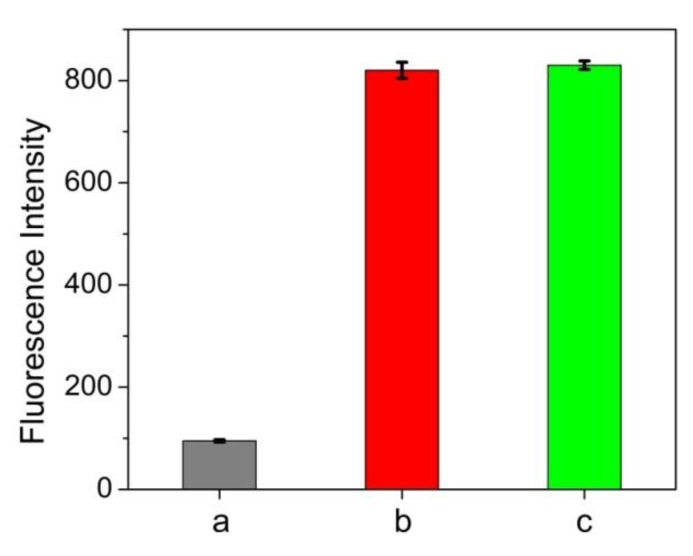
Fluorescence intensities of (a) 0.5 mg/mL ATT-AuNCs, (b) 0.5 mg/mL ATT-AuNCs + 500 μg/mL HSA, and (c) 0.5 mg/mL ATT-AuNCs + 500 μg/mL BSA. Error bars represent the standard deviations across three repetitive experiments.

In order to elucidate the practical feasibility of the approach, a variety of biological samples were determined by the proposed method and compared with classical BCA method. As we know, it is of great importance to make a quick determination of total protein in plasma or serum content which is closely related to various diseases such as hypoalbuminemia [29] and hypertensive disease [30]. Figure 5 showed that human serum albumin (HSA) can also induce fluorescence enhancement of ATT-AuNCs and its efficiency is comparable to that of BSA. This result confirms that ATT-AuNC possesses the capacity to bind HSA. As shown in Table 2 and Table 3, the results of total protein content measured by this method agree well with those obtained by the standard BCA method (Table 2 and Table 3). In addition, we used BSA as a standard agent to conduct recovery experiments in human plasma samples. It was found that the recoveries were observed to range from 104% to 112%. Therefore, the proposed protein assay yields satisfactory results for total protein determination in human plasma and serum samples.

### 3.4. Determination of Proteins in Milk Samples

Food safety problems caused by the melamine incident have affected the entire food industry [31]. It can lead to different degrees of kidney failure or death, and induce bladder stones and urinary system diseases [32]. Classic food proteins determination (Kjeldahl methods) is cumbersome, time-consuming, and has a lack of specificity [33]. Thus, the demand for a rapid, accurate milk protein assay has increased dramatically. In this study, we chose three brands of pure milk in the supermarket randomly for protein assay. The results of total protein content obtained by the proposed method agree well with those measured by the BCA method (Table 4).

### 3.5. Determination of Cellular Total Protein Concentration

Various kinds of cell protein factors were involved in complex biochemical processes like inflammatory responses, tumor growth, cell apoptosis and angiogenesis [34,35]. Thus, assay of cell total protein is of great significance. In the present study, we monitored cell total protein. HL 60 was used as a model cell. The results of total protein content obtained by the proposed method agree well with those measured by the BCA method (Table 5). It implies this method has potential application in the detection of total cellular protein.

## 4. Conclusions

Herein, we present the BSA induced fluorescence enhancement of ATT-AuNCs through protein–nanocluster interactions, and it was successfully used to determine the total protein content of various biological samples. Fluorescence spectroscopy studies shed some light on the underlying mechanism of fluorescence enhancement of AuNCs upon protein adsorption. It turned out that the dominating quenching mechanism of protein–nanocluster interactions was the static quenching process. The experimental results indicate that this new method is better than the traditional BCA method in its compatibility with high concentrations of reducing sugar, which proposed an alternative scheme to determine the total protein content.

## Figures and Tables

**Table 1:** Calculated thermodynamic parameters for ATT-AuNCs-BSA composite.

T (°C)	K (10^8^ L mol^−1^)	ΔH (kJ mol^−1^)	ΔG (kJ)	ΔS (J mol^−1^ K^−1^)	R	SD
21	5.70	17.583	−49.275	227.380	0.9996	0.97
33	7.38		−51.948		0.9978	2.41
38	8.51		−53.167		0.9997	0.70

**Table 2:** The analytical results of plasma total protein.

Sample	Proposed Method (mg/mL, n = 3)	BCA Method (mg/mL, n = 3)	F-Test ^1^	*t*-Test ^1^
1	62.60 ± 1.35	62.84 ± 1.27	1.13	0.22
2	61.73 ± 0.28	62.20 ± 1.17	17.46	0.68
3	58.08 ± 1.15	58.55 ± 1.26	1.21	0.48
4	58.95 ± 0.73	58.05 ± 0.78	1.14	1.46
5	65.18 ± 2.62	65.95 ± 1.42	3.40	0.45
6	63.26 ± 1.88	64.80 ± 1.66	1.28	1.07

^1^ F_0.05, 2, 2_ = 19.00, t_0.05, 4_ = 2.776.

**Table 3:** The results of serum total protein.

Sample	Proposed Method (mg/mL, n = 3)	BCA Method (mg/mL, n = 3)	F-Test ^1^	*t*-Test ^1^
1	59.15 ± 0.82	61.25 ± 2.78	11.49	1.26
2	65.28 ± 1.63	64.86 ± 1.96	1.45	0.29
3	64.81 ± 1.45	65.02 ± 0.52	7.78	0.24
4	70.56 ± 0.48	71.74 ± 1.93	16.17	1.03

^1^ F_0.05, 2, 2_ = 19.00, t_0.05, 4_ = 2.776.

**Table 4:** The results of milk total protein.

Sample	Proposed Method (mg/mL, n = 3)	BCA Method (mg/mL, n = 3)	F-Test ^1^	*t*-Test ^1^
1	31.17 ± 0.18	32.29 ± 0.43	5.71	4.16
2	32.44 ± 0.19	32.01 ± 0.52	7.49	1.34
3	30.01 ± 0.16	30.05 ± 0.50	9.77	0.14

^1^ F_0.05, 2, 2_ = 19.00, t_0.05, 4_ = 2.776.

**Table 5:** The results of cell total protein.

Sample	Proposed Method (mg/mL, n = 3)	BCA Method (mg/mL, n = 3)	F-Test ^1^	*t*-Test ^1^
1	5.52 ± 0.034	5.51 ± 0.026	1.71	0.40
2	6.77 ± 0.046	6.84 ± 0.072	2.45	1.42

^1^ F_0.05, 2, 2_ = 19.00, t_0.05, 4_ = 2.776.
